# Obituary for Professor Yoel Kloog

**DOI:** 10.1038/s41420-020-0254-7

**Published:** 2020-04-20

**Authors:** Ronit Haklai, Reuven Stein

**Affiliations:** Department of Neurobiology, School of Neurobiology, Biochemistry and Biophysics, George S. Wise Faculty of Life Sciences, Tel Aviv University, Ramat Aviv, 69978 Israel

**Keywords:** Cancer, Oncogenes

**Yoel Kloog (1948–2019)**
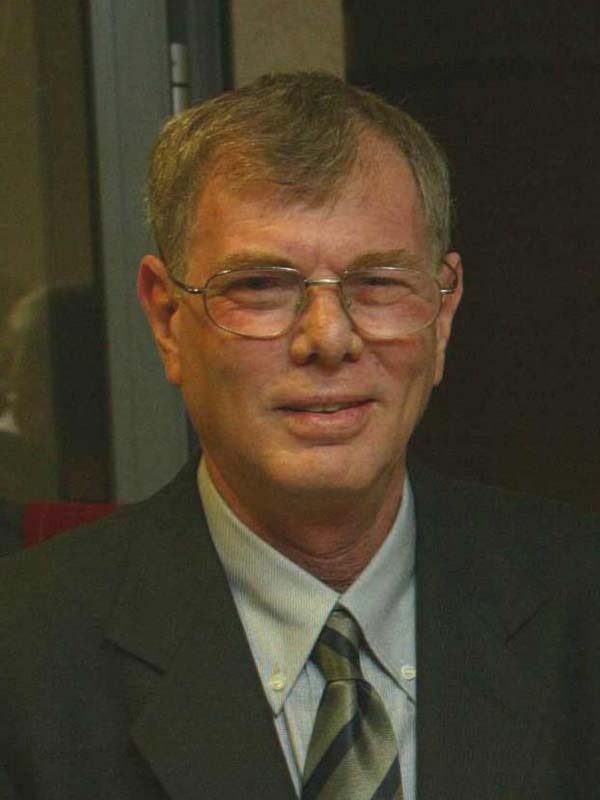


A biochemist whose discoveries have led to a better understanding of Ras function in health and disease.

Yoel Kloog was a passionate researcher of signaling in normal and diseased cells. His research has significantly contributed to the development of new concepts in signal transduction and to the design of potential therapeutic modalities for the treatment of severe diseases, including cancer, neurological disorders, such as head trauma and stroke, and immune-related diseases, including multiple sclerosis, lupus, restenosis, and atherosclerosis.

Yoel Kloog was born in Israel in 1948. He pursued a bachelor of science degree in biology and master and PhD degrees in biochemistry at Tel Aviv University. He then continued for postdoctoral training at the National Institute of Mental Health (Bethesda, MD), under the supervision of the Nobel Laureate Julius Axelrod, a renowned expert in biochemical pharmacology. He started his own lab at Tel Aviv University in 1982. Kloog was not only a great scientist, but also a leader and educator. He served as the Dean of the George S. Wise Faculty of Life Sciences at Tel Aviv university for 7 years. During this time, the faculty has made significant progress on all fronts, from recruitment of excellent new researchers to obtaining support for the Graduate School and the development of the interdepartmental equipment facilities.

Over the years, the research of Yoel Kloog has focused on two main avenues. The first is cell signaling, which attracted his interest from the level of cell surface receptors to intracellular signal transduction. The second avenue to which he devoted major efforts was drug development and therapies.

In the cell-signaling aspect of his research, Yoel Kloog has studied the muscarinic acetylcholine and the sarafotoxins/endothelin receptors, as well as Ras signaling. When he first embarked on studying the muscarinic acetylcholine receptor, it was more of a concept than an established receptor. Using radio-labeled agonist and antagonist ligands, Kloog characterized the biochemical properties of the receptor and established its expression in different tissues and its connection to biological effects, such as aging and adaptation to heat. He also studied the relationship between the muscarinic receptor and guanine nucleotides, as well as its interaction with sodium channels. These seminal studies have helped to establish the functional importance of the muscarinic receptors.

Among his important discoveries in cell signaling was the identification of the sarafotoxins (SRTXs), a group of toxins present in the venom of the *Atractaspis engaddensis* snake from Israel Jordan Valley, as compounds that bind to the endothelin (ET) receptor. Kloog was able to elucidate the signaling pathways initiated by these receptors, inducing vasoconstriction. Furthermore, the unusually high degree of sequence homology between the SRTXs and the mammalian ET vasoconstrictor peptides, led Kloog and his colleagues to suggest that the endothelins are the endogenous ligands for the same receptors that bind SRTXs. This notion was confirmed only later, when the mammalian ET receptor was identified and cloned by others.

In the last 25 years, Kloog focused on studying the Ras oncoproteins. This research has begun when he observed that Ras is carboxymethylated by a membrane-associated methylation enzyme. This observation has led him to design and prepare new inhibitors of the enzyme, in order to inhibit Ras-dependent growth of cancer cells. It turned out that cell growth by these compounds was inhibited not only by inhibition of Ras methylation, but also by a novel mechanism where the inhibitors detached the lipid-modified (prenylated) active Ras protein from the cell membrane. These studies led him to envision “prenyl-binding domains in Ras interacting proteins” and then to identify galectin-1 and galectin-3 as such Ras-binding partners. The latter studies demonstrated a novel mechanism whereby galectin-1 and galectin-3 control both the duration and selectivity of H-Ras and K-Ras oncogenic signals, respectively. The idea that the farnesyl (a lipid prenyl group) of Ras might play a functional role in Ras signaling through its interactions with Ras-binding partners was a totally new concept. Following extensive experimentation in his laboratory and in collaboration with world-renowned scientists from a wide range of disciplines, this new concept was confirmed.

Yoel Kloog also had a passion for applied biology. He used his vast knowledge in biochemistry and molecular pharmacology to design drugs for new therapeutic modalities. In his early studies, he focused on the NMDA/glutamate receptors where he developed pharmacological approaches to block the receptor. These studies led to the discovery that the cannabinoid derivative HU-211 is a noncompetitive NMDA/glutamate receptor blocker that can inhibit glutamate neurotoxicity. HU-211 was later tested in clinical trials for treatment of severe closed head injury (phase II).

The unique family of Ras inhibitors prepared by Kloog’s group have not only helped to unravel complex aspects of Ras signal transduction, but also have great potential for use in the treatment of many types of human diseases where chronically active Ras is involved. Farnesyl thiosalicylic acid (FTS, United States Adapted Name Council approved the name “Salirasib”), the lead compound, was in clinical trials. After completing Phase I safety trials at MD Anderson Cancer Center, Salirasib has entered Phase II trials at Johns Hopkins Medical Center (for pancreatic cancer) and Memorial Sloan Kettering, NY (for lung cancer) as a single drug treatment. Unfortunately, the clinical trials were stopped because of Kloog’s illness.

The significant contribution of Yoel Kloog’s research was recognized worldwide. He was invited to contribute reviews and to deliver talks in many international meetings. Moreover, his papers were frequently the subjects of special commentaries, highlights, and cover pages. Many of his 250 publications in leading peer-reviewed international journals are considered standard references and are frequently cited. In addition, he has submitted 37 drug patents.

Yoel Kloog loved his lab work and had an almost superhuman work ethic. He worked 7 days a week about 12 h per day and never complained. Besides being a great scientist, Kloog was also an excellent teacher and mentor. He loved his students and encouraged them to think outside of the box, to ask questions, and gave them immense freedom. Indeed, the record of his students indicates that they have been inspired by him, and many of them followed his footsteps and became excellent biomedical researchers and teachers. Some went on to faculty positions in Israel. In this regard, it is worth noting what he wrote in one of his letters: “I should say that much as I may have played some part in inspiring their scientific and medical careers, I myself continue to be inspired by the motivation of these highly talented individuals. The young generation of my present students hold promise that they will do even better.”

In 2008, Kloog was diagnosed with lung cancer. However, he was a fighter and an endless optimist, and did not let his illness to affect his regular life. Despite the harsh treatments, he continued to work every day, write grant applications and papers, and manage the faculty as a Dean. Although Kloog miraculously was cured from the cancer, the harsh treatments took their tolls, and despite his courageous fight, he died on the 21st of October 2019 from the complications of the treatments.

Yoel Kloog was a great family person. Despite his great scientific achievements and special position as a faculty dean, he was very modest and treated equally with great respect and loved everybody, cleaning people, janitors, students, and PIs.

He has left behind a wife, three children, seven grandchildren, and many generations of students and researchers who are learning to cope with his loss.

Life is like riding a bicycle. To keep your balance, you must keep moving—*Albert Einstein*

